# In regard to “Tran A, Zhang J, Woods K, Yu V, Nguyen D, Gustafson G, Rosen L, Sheng K. Treatment planning comparison of IMPT, VMAT and 4π radiotherapy for prostate cases. Radiation oncology. 2017 Jan 11; 12(1):10”

**DOI:** 10.1186/s13014-018-1009-y

**Published:** 2018-04-12

**Authors:** Biplab Sarkar

**Affiliations:** Department of radiation Oncology, Fortis Memorial Research Institute, Gurgaon, India

**Keywords:** 4π radiotherapy, π, Sold angle, Solid geometry, 3D Euclidian space, Gantry, Couch, Linear accelerator, Radian

## Abstract

This article describe the three dimensional geometrical incompetency of the term “4π radiotherapy”; frequently used in radiation oncology to establish the superiority (or rather complexity) of particular kind of external beam delivery technique. It was claimed by several researchers, to obtain 4π^c^ solid angle at target centre created by the tele-therapy delivery machine in three dimensional Euclidian space. However with the present design of linear accelerator (or any other tele-therapy machine) it is not possible to achieve more than 2π^c^ with the allowed boundary condition of 0 ≤ Gnatry position≤π^c^ and $-\frac{\pi^c}{2}$≤Couch Position≤$+\frac{\pi^c}{2}$ .

This article describes why it is not possible to achieve a 4π^c^ solid angle at any point in three dimensional Euclidian spaces. This article also recommends not to use the terminology “4π radiotherapy” for describing any external beam technique or its complexity as this term is geometrically wrong.

## Text

I would like to make a comment on the “4π radiotherapy”; mentioned by Tarn et al. regarding the 4π radiotherapy for prostate cases. The concept of 4π radiotherapy was originally floated by Dong et al. *in 2013;* and subsequently used by several authors; calming to have delivered a radiotherapy technique which look into a tumour from 4π solid angle [1–8].

The geometrical constriction of a teletherapy machine/linear accelerator mechanically represent a Cantilever, where head anchored at only one end with a vertical support from which it is protruding. A teletherapy machine having two additional degree of freedoms; a full arc gantry rotation of (0-2π^c^) and a half arc couch rotation (0-π^c^).

Geometry of three dimensional Euclidian space, solid geometry, defines the angle obtained by a surface in terms of solid angle presented as following.

$$ \mathrm{d}\Omega =\frac{ds}{r^2} $$

where ds is the surface area and r is the radius vector can obtained a solid angle of 4π^c^ at its centre as described below.

Solid angle at the centre of a sphere

$$ \Omega =\frac{\mathrm{Area}}{r^2}=\frac{1}{r^2}\left[{\int}_{\theta =0}^{\pi }{\int}_{\upvarphi =0}^{2\pi}\left( rSin\theta d\varphi \right).\left( rd\theta \right)\right] $$

where, r, θ and φ are radius vector polar and azimuthal angle.

$$ =\frac{4{\pi r}^2}{r^2}=4{\uppi}^{\mathrm{c}}\ \left[={12.56}^{\mathrm{c}};{}^{\mathrm{c}}\ \mathrm{is}\ \mathrm{steradian}\right]. $$

Under the geometrical boundary condition of the linear accelerator rotational degree of freedom (gantry: 0^0^–360^0^-0^0^ and couch 90^0^–0^0^-270^0^; however 90^0^–180^0^–270^0^ is inaccessible to couch) azimuthal angle integration reduces to 0-π^c^. Therefore maximum accessible solid angle for a linear accelerator machine is

$$ =\frac{1}{r^2}{\int}_{\theta =0}^{\pi }{\int}_{\upvarphi =0}^{\pi}\left( rSin\theta d\varphi \right).\left( rd\theta \right)=2{\uppi}^{\mathrm{c}};\mathrm{solid}\ \mathrm{angle}\ \mathrm{obtained}\ \mathrm{by}\ \mathrm{a}\ \mathrm{hemisphere}. $$

This type of hemispherical therapy delivery is only possible for two ends of the human that is either brain or foot. Rest of the length (head neck-thorax-abdomen-pelvis) of the human body is not accessible even for a 2π^c^ radiotherapy. Therefore claimed to have “4π radiotherapy” for prostate does not hold geometrically.

I would like to mention that, as an example, the solid angle created by a full arc (0-2π^c^) gantry rotation with a 40 × 40 cm^2^ field opening and couch angle at zero degree is

$$ \Omega\ \mathrm{Full}\ \mathrm{ARC}=\frac{Area}{r^2}=\frac{1}{100\ {cm}^2}\ \left[2\uppi\ 100\ \mathrm{cm}\times 40\ \mathrm{cm}\right]={2.51}^{\mathrm{c}}, $$

which is (1/5)^th^ of the 4π^c^. Solid angle further reduces with the multileaf collimator shaped or blocked fields.

To perform a “4π radiotherapy” a patient need to be point and radiotherapy machines should be able to move to any point on the surface of a spare; under the present design of any teletherapy machines like linear accelerator, tele-cobalt, tomotherapy (Accuray Inc., Madison, WI) or Cyber knife (Accuray Inc., Madison, WI) cannot perform a “4π radiotherapy”. Probably only Brachytherapy can be near to a “4π radiotherapy” approximating (highly) the source as a point source.

A generalised geometrical misconception of “4π radiotherapy” was floated in 2013 by Dong et al. and propagating up to date (Victoria et al.) [1–8].

The technique described by the listed authors in this article could have been identified (or nomenclated) by something else but definitely not by “4π radiotherapy”. “4π Radiotherapy” is a geometrically non-viable and scientifically wrong concept; tagged with a fancy name to establish its superiority over generalized non-coplanar technique. Therefore the misconception about “4π radiotherapy” need to be corrected should not be used in future.

## Abbreviations

^c^: Steradian or radian unit of solid angle
r, θ and φ: Radius vector polar and azimuthal angle in spherical polar coordinate
π: Pi is a number - approximately 3.142
Ω: Solid angle: defined as ratio between area and squire of the radius vector
